# Newly Synthesized APOBEC3G Is Incorporated into HIV Virions, Inhibited by HIV RNA, and Subsequently Activated by RNase H

**DOI:** 10.1371/journal.ppat.0030015

**Published:** 2007-02-09

**Authors:** Vanessa B Soros, Wes Yonemoto, Warner C Greene

**Affiliations:** 1 Gladstone Institute of Virology and Immunology, San Francisco, California, United States of America; 2 Department of Medicine, University of California San Francisco, San Francisco, California, United States of America; 3 Department of Microbiology and Immunology, University of California San Francisco, San Francisco, California, United States of America; National Institutes of Health, United States of America

## Abstract

APOBEC3G (A3G) is a potent antiretroviral deoxycytidine deaminase that, when incorporated into HIV virions, hypermutates nascent viral DNA formed during reverse transcription. HIV Vif counters the effect of A3G by depleting intracellular stores of the enzyme, thereby blocking its virion incorporation. Through pulse-chase analyses, we demonstrate that virion A3G is mainly recruited from the cellular pool of newly synthesized enzyme compared to older “mature” A3G already residing in high-molecular-mass RNA–protein complexes. Virion-incorporated A3G forms a large complex with viral genomic RNA that is clearly distinct from cellular HMM A3G complexes, as revealed by both gel filtration and biochemical fractionation. Unexpectedly, the enzymatic activity of virion-incorporated A3G is lost upon its stable association with HIV RNA. The activity of the latent A3G enzyme is ultimately restored during reverse transcription by the action of HIV RNase H. Degradation of the viral genomic RNA by RNase H not only generates the minus-strand DNA substrate targeted by A3G for hypermutation but also removes the inhibitory RNA bound to A3G, thereby enabling its function as a deoxycytidine deaminase. These findings highlight an unexpected interplay between host and virus where initiation of antiviral enzymatic activity is dependent on the action of an essential viral enzyme.

## Author Summary

APOBEC3G (A3G) is a cellular enzyme that promotes DNA mutagenesis and can restrict infection by HIV-1. However, HIV counters the antiviral effects of A3G through the action of its Vif protein. In the absence of Vif, A3G is effectively incorporated into virions, where it mutagenizes the first DNA copy (cDNA) generated during reverse transcription of the viral RNA genome. A3G also appears to be able to inhibit HIV via nonenzymatic mechanisms. A3G and related deoxycytidine deaminases can also inhibit the growth of retroviruses other than HIV and protect the cellular genome from endogenous mobile retroelements. In this study, we analyzed the recruitment and enzymatic activity of A3G incorporated into HIVΔVif virions. Unexpectedly, we found that the binding of A3G to viral genomic RNA led to inactivation of the enzyme. However, latent A3G was ultimately activated through the action of HIV RNase H, which degrades the RNA genome during reverse transcription. These findings highlight an unexpected interplay between a host enzyme and HIV, where the antiviral enzymatic activity of the host factor (A3G) is dependent on the action of an essential HIV enzyme (RNase H). The strong interaction with viral RNA also suggests a potential mechanism by which A3G could exert antiviral activity in the absence of enzymatic activity, by physically impeding reverse transcription.

## Introduction

APOBEC3G (A3G) is a highly active antiretroviral deoxycytidine deaminase that greatly impairs HIV spread in cultures of activated CD4 T cells provided the HIV Vif protein is absent [1]. In these activated cells, the antiviral action of A3G involves its effective incorporation into budding virions and subsequent hypermutation of nascent viral DNA formed during the next round of infection [2–6]. Vif has been proposed to block the incorporation of A3G into HIV virions by targeting this enzyme for accelerated degradation in the 26S proteasome [7–12] and partially blocking its de novo synthesis [7,13]. A different situation occurs in resting CD4 T cells and likely monocytes, which are not permissive for HIV infection. In these cells, a low-molecular-mass (LMM) form of cellular A3G is present, and it functions as a potent postentry restriction factor for HIV by blocking late reverse transcription [14]. This antiviral action of A3G is unchecked by Vif because insufficient quantities of Vif are present in the incoming virions and the virus has not progressed far enough into its life cycle to synthesize new Vif. Thus, the growth of wild-type (WT) HIV is effectively restricted in these cells by LMM A3G.

Incorporation of A3G into virions budding from HIV-infected CD4 T cells has been proposed to involve assembly with the nucleocapsid (NC) component of the Gag polyprotein and/or viral genomic RNA [15–22]. Recent studies with highly divergent Gag proteins [23] or treatment with RNase A [16,18,19,22] suggest that Gag binding may be indirect, involving an RNA intermediate. Following the entry of A3G-containing virions into new target cells, A3G deoxycytidine deaminase activity targets the minus-strand DNA product of reverse transcription, leading to the appearance of deoxyuridines in lieu of deoxycytidines at canonical sites of deamination (5′C*C*; the residue targeted for A3G-mediated deamination is italicized) [1–6,24]. The nontemplated action of various DNA repair enzymes, including uracil *N*-glycosylase, may mediate DNA strand cleavage [25], although a recent study suggests that uracil-*N*-glycosylase 2 is dispensable for the antiviral action of A3G [26]. If plus-strand synthesis proceeds, dA residues are introduced at sites of dC deamination, which results in dG-to-dA hypermutation in the viral coding strand. These mutations may compromise HIV infectivity by altering various viral open reading frames and introducing inappropriate translation termination codons.

In contrast to the LMM form of A3G in resting CD4 T cells, A3G in activated CD4 T cells principally resides in high-molecular-mass (HMM) RNA–protein complexes [14]. These complexes include Staufen RNA transporting granules and Ro/La ribonucleoprotein (RNP) complexes containing Alu and hY retrotransposon RNA [27,28]. These complexes lack detectable deoxycytidine deaminase activity in vitro but interrupt Alu retrotransposition by sequestering the retroelement RNA in the cytoplasm away from the requisite nuclear LINE machinery. Treatment of these complexes with RNase A promotes complex disassembly and generates the LMM, enzymatically active form of A3G. Thus, the cellular forms of A3G in resting and activated CD4 T cells are remarkably different. The recruitment of A3G into HMM RNA–protein complexes during the course of T-cell activation likely explains why cellular A3G fails to function as a postentry restriction factor for HIV in these activated cells.

The purposes of this study were to analyze the form of A3G that is incorporated into HIVΔVif virions and to assess its enzymatic activity. Since virion A3G is readily able to mediate hypermutation of viral DNA formed during reverse transcription, we anticipated that enzymatically active forms of A3G would predominate in virions. We have found that newly synthesized A3G, not preexisting A3G already assembled into the inactive cellular HMM complexes, is encapsidated into budding virions. We also found that A3G recruited into virions assembles with viral RNA to form a large intravirion A3G complex (IVAC) that is enzymatically inactive. Finally, we have demonstrated that the action of viral RNase H during reverse transcription ultimately releases A3G from its state of inhibition, allowing hypermutation of the minus-strand viral DNA. Thus, activation of the enzyme-dependent antiviral action of A3G appears to critically depend on the action of an HIV enzyme, RNase H.

## Results

### A3G Recruited into Virions Cofractionates with Virion Cores

**Figure 1:**
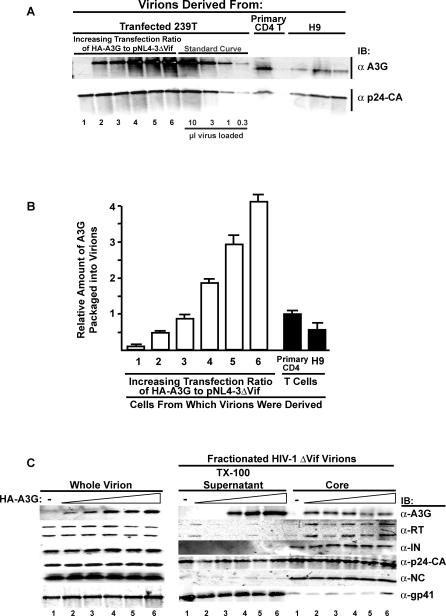
A3G Is Incorporated into Virion Cores but A3G Overexpression in Cells Results in Additional A3G Packaging Outside of the Core (A) HA-A3G–containing ΔVif virions were generated from 293T cells transfected with a fixed amount of proviral plasmid (60 μg) and increasing doses of HA-A3G (0 to 20 μg). Empty HA vector (0 to 20 μg) was used as balance DNA in the transfections. Sample number 1 = 0 (μg of HA-A3G):60 (μg of pNL4-3ΔVif), 2 = 1:60, 3 = 2:60, 4 = 5:60, 5 = 10:60, 6 = 20:60. ΔVif virions were also derived from the H9 T cell line and primary CD4 T cells, which endogenously express A3G. The virion lysates were subjected to immunoblotting with antibodies specific for p24-CA and A3G. The immunoblot is representative of several independent analyses used to generate the graph in (B). (B) Graphical representation of quantification from immunoblots in (A) and unpublished data. Data are averaged from three independent transfections of 293T cells, five independent spinoculations of activated primary CD4 T cells, and three independent spinoculations of H9 T cells. The error bars represent standard deviation. The relative ratio of packaged A3G to p24-CA is plotted, with virions derived from CD4 T cells assigned a value of 1. (C) Virions containing increasing amounts of HA-A3G relative to p24-CA were solubilized by brief Triton X-100 treatment to generate virion cores containing p24-CA, IN, RT, and NC and supernatants containing gp41 and p24-CA. The triangles represent the increasing dose of A3G and correspond exactly to the numbered samples presented in Figure 1A. The immunoblots (IB) were also probed for A3G to determine the amount packaged into virion cores.

Initially, we sought to identify transfection conditions for the generation of A3G-containing HIV virions that would recapitulate the virion encapsidation of A3G that naturally occurs in T cell lines and primary T cells infected with HIVΔVif viruses. Activated blood-derived primary CD4 T cells or H9 T cells expressing endogenous A3G were spinoculated with HIVΔVif and emergent viruses were harvested 2 d postinfection. In parallel, 293T cells were cotransfected with a fixed dose of proviral expression plasmid DNA and increasing doses of A3G expression plasmid DNA. Virions were similarly collected from the transfected cells 2 d later, after purification of virions by ultracentrifugation through iodixanol cushions. Virion lysates were subjected to immunoblotting to determine the amount of A3G incorporated relative to p24-CA content. These virion preparations were not contaminated with significant cellular material or microvesicles, as determined by immunoblotting with anti–14-3-3γ and anti-CD45 antibodies (Figure S1). Transfection of increasing amounts of A3G expression plasmid resulted in increasing amounts of A3G virion incorporation relative to p24-Capsid (CA) (Figure 1A and 1B). However, when compared to virions produced from infected primary CD4 T cells or H9 T cells, the 293T transfection conditions that best recapitulated the “natural” packaging levels of endogenously expressed A3G were achieved at a plasmid microgram ratio of 2 (HA-A3G) to 60 (pNL4–3ΔVif), which equals a molar ratio of 1 (HA-A3G) to 12.5 (pNL4–3ΔVif). Thus, this condition was used for the production of A3G-containing virions in all the subsequent experiments unless otherwise indicated.

To determine if A3G is packaged into the core of HIV virions and to assess the localization of the additional A3G packaging that occurred at higher transfection doses, virions were subjected to biochemical fractionation. The virion envelope was removed by brief solubilization with Triton X-100, as previously described [29], yielding separable virion cores containing p24-CA, integrase (IN), reverse transcriptase (RT), and NC (Figure 1C). Fractionation of viruses containing A3G at levels comparable to those of virions budding from primary CD4 T cells revealed that A3G is indeed packaged into virion cores (Figure 1C). However, virions derived from cells expressing higher levels of A3G (for example, 20 μg of HA-A3G:60 μg of pNL4–3ΔVif) packaged a similar amount of A3G into virion cores as well as additional A3G that fractionated into the gp41-containing supernatant after Triton X-100 solubilization (Figure 1A–1C). These findings suggest that when A3G is overexpressed in virus-producing cells, considerable amounts of the enzyme reside outside the viral core, likely in the viral matrix region, between the core and outer envelope (for example, 1:3 μg ratio). The appearance of approximately half the virion p24-CA in the Triton X-100 supernatant after solubilization similarly reflects excess Gag packaged into virions that does not contribute to core formation [30]. Thus, A3G may gain access to HIV virion cores through a specific interaction with viral RNA and/or Gag (NC). However, under conditions of overexpression, a lower-affinity interaction, perhaps directly between A3G and Gag, results in the recruitment of additional enzyme into the detergent-sensitive matrix space, into which excess Gag is also packed [30].

### Newly Translated A3G Is Incorporated into HIV Virions

**Figure 2:**
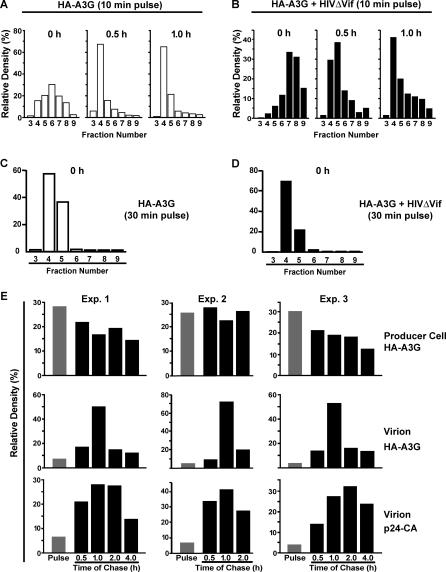
Newly Synthesized HA-A3G Is Recruited into HIV-1 Virions (A) Cells transfected with HA-A3G were pulse radiolabeled for 10 min, chased with cold medium, and harvested at 0, 0.5, and 1 h. Chase lysate was subjected to Sepharose CL-6B gel filtration (10 fractions collected per lysate), HA-A3G was immunoprecipitated from each fraction, and the relative amount of radiolabeled HA-A3G in each fraction was determined by autoradiography. Fraction 4 corresponds to HMM complexes, and fractions 6 to 8 contain proteins similar in size to LMM A3G. See Figure S2A for Sepharose CL-6B fractionation performance. (B) Cells transfected with HA-A3G and pNL4–3ΔVif were assessed as in (A). (C) Cells transfected with HA-A3G were assessed as in (A), except that the pulse was extended to 30 min. Plotted is the *t* = 0-h pulse sample. (D) Cells transfected with HA-A3G and pNL4–3ΔVif were assessed as in (C). (A–D) Data shown are representative of three independent experiments for each panel. (E) Virus-producing cells were pulse radiolabeled and chased with cold medium, and both cells and virus-containing supernatants were collected at 0.5, 1, 2, and 4 h. As all of the supernatant was collected at the indicated time points, the virions harvested represent only those that budded during the intervening time period. Plotted is the percent density of the immunoprecipitated protein for a given time point relative to the total radioactive density of all time points. Upper panels, immunoprecipitates of HA-A3G from the producer cells. Middle panels, immunoprecipitates of HA-A3G from virions. Lower panels, immunoprecipitates of p24-CA from virions. Data are from three experiments performed independently.

Under steady-state conditions in activated CD4 T cells, cellular A3G resides in an HMM RNase A–sensitive complex of 5 to 15 MDa [14,27]. The observation that HA-A3G incorporated into virions packages into the virion core suggested several possible cellular sources of the enzyme. The first possibility, albeit unlikely, is that entire 5- to 15-MDa cellular A3G complexes are recruited into the virion core. Alternatively, HIV RNA and Gag may promote release of A3G from the cellular HMM A3G complex, allowing its recruitment into the virion, with or without a limited subset of cellular cofactors, as has been suggested [28]. Finally, newly synthesized LMM A3G not yet assembled with cellular cofactors or RNA may be recruited into the virion through its association with viral RNA and Gag. To determine if newly synthesized A3G, more “mature” A3G, or both serve as cellular reservoirs for virion recruitment, we performed pulse-chase radiolabeling studies. First, the time course for recruitment of newly synthesized HA-A3G into cellular HMM complexes in the absence of proviral gene expression was determined. Cells were pulsed with radiolabel for 10 min, followed by chases of 30 min to 3 h. Size-fractionation (Figure S2A) of the pulse and chase lysates identified the presence of pulse-labeled HA-A3G initially in low and intermediate mass fractions (Figure 2A, *t* = 0 fractions 6 and 7) that was chased into HMM complexes within 30 min (Figure 2A, A3G in fractions 4 and 5 at 0.5 and 1 h). The radiolabeled A3G remained stably associated with the cellular HMM complex during longer chase periods (Figures 2A and S2B). Thus, newly synthesized A3G is initially LMM and recruited within 30 min into stable cellular HMM RNA–protein complexes. When HIVΔVif was coexpressed, we observed that the presence of viral RNA and proteins did not alter the ability of newly synthesized LMM A3G to assemble into cellular HMM RNA–protein complexes (Figure 2B).

To assess whether newly synthesized or more “mature” preexisting cellular HMM A3G is recruited into virions, we performed similar pulse-chase radiolabeling studies in cells producing HIV. To enhance radiolabeling and detection of intravirion A3G, the length of the pulse was extended from 10 min to 30 min. The longer pulse time did not affect A3G assembly into cellular HMM complexes, in either the absence (Figure 2C) or presence (Figure 2D) of HIVΔVif expression; however, it did mask the chase of LMM A3G into HMM A3G. Expression of HIVΔVif also did not affect the turnover of radiolabeled A3G (Figure S2D). Both producer cells and their supernatants containing virions were collected and analyzed simultaneously for radiolabeled HA-A3G and p24-CA content. Since all of the virus-containing supernatants were collected at the indicated time points, the radiolabel present in virion p24-CA or HA-A3G reflects labeling events occurring during the discrete intervening time points. In each of the three independent experiments shown, incorporation of radiolabeled p24-CA into virions increased with increasing chase time over the first 1 to 2 h and then declined by 4 h (Figure 2E, lower panels). The incorporation pattern of p24-CA over time is consistent with previous reports [31,32] showing increased accumulation of radiolabeled p24 in virions with long cumulative chase times. In contrast, HA-A3G incorporation into virions displayed a sharp spike between 30 and 60 min after the pulse (Figure 2E, middle panels), even though large cellular pools of radiolabeled A3G were present both before and after this time point (Figure 2E, upper panels). Specifically, despite the presence of radiolabeled cellular HA-A3G at the 2- and 4-h time points, these pools of A3G were not effectively incorporated into virions compared to the 1-h time point. The distinct peak of incorporation of radiolabeled A3G at 1 h after pulse also was not due to a relative loss of radiolabeled cellular A3G available for virion incorporation at the later collection times, since normalization by the available radiolabeled pool of cellular enzyme did not alter the distinct early kinetic pattern for A3G incorporation into virions (Figure S2C). These findings indicate that newly synthesized A3G less than 1.5 h old is incorporated HIV virions and that older, more “mature” A3G in HMM complexes is apparently less available for virion incorporation during the time course examined here. Using a modified version of these experiments (infection with HIVΔVif instead of transfection of a proviral DNA plasmid) and extended chase times, we observed a similar trend of newly synthesized radiolabeled A3G incorporation into virions and low-to-undetectable levels of radiolabeled A3G in virions up to 9 h after the pulse period (Figure S2E).

### A3G Assembles into a Large RNA-Containing Intravirion Complex (Intravirion A3G Complex)

**Figure 3:**
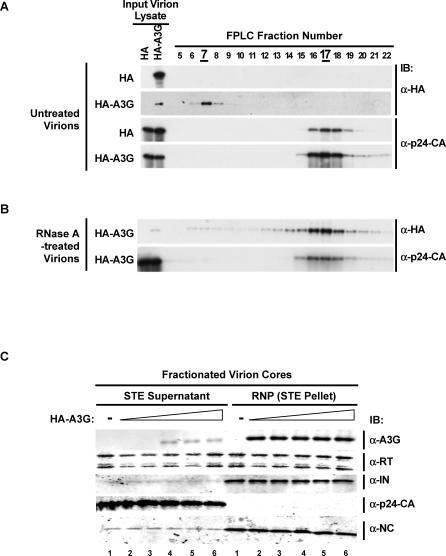
Virion-Incorporated HA-A3G Resides in a Large RNase A–Sensitive Complex and Biochemically Fractionates with Viral RNP Proteins (A) Virions collected from cells expressing HIV-1ΔVif contain HA-A3G that predominantly fractionates in a large complex (fractions 6 to 8) as assessed by gel filtration. (B) The IVAC is sensitive to RNase A treatment which shifts HA-A3G into lower fractions (fractions 15 to 19). (C) Virion cores obtained in Figure 1 were subjected to further biochemical fractionation to generate viral RNPs. Shown are the viral RNPs from virions either lacking or containing A3G, as indicated, and containing viral RT, IN, and NC but not p24-CA, as detected by immunoblotting (IB). The triangles represent the increasing dose of A3G relative to provirus and correspond exactly to the sample numbers in Figure 1A.

The finding that newly synthesized A3G is packaged into virion cores (Figure 1C) coupled with the observation that newly synthesized A3G rapidly forms HMM complexes in cells (Figure 2A–2D) led us to next examine whether intravirion A3G resolves as monomers/dimers or instead as a larger complex. We hypothesized that A3G in virions might remain in an enzymatically active LMM form, because it ultimately deaminates the viral minus-strand DNA synthesized during reverse transcription. Lysates derived from virions containing HA-tagged A3G were size-fractionated by fast protein liquid chromatography (FPLC). Each fraction was then analyzed by SDS-PAGE and immunoblotting with anti-HA monoclonal antibodies (Figure 3A). Surprisingly, virion-incorporated HA-A3G was detected almost exclusively in the HMM region, eluting in the void volume of the Superose 6 column (Figure 3A). This result was not due to incomplete lysis of the virion cores since the p24 viral capsid (p24-CA) was detected only in the expected LMM fractions.

To determine if the IVAC contains an RNA component, virion lysates were treated with RNase A (Figure 3B). Under these conditions, HA-A3G shifted to LMM fractions consistent in size with monomers and/or dimers of the enzyme. Therefore, reminiscent of the cellular forms of A3G present in activated CD4 T cells [14] (and in HIV-producing cells, as shown below), A3G incorporated into virion assembles into large RNA–protein complexes that are distinct from the cellular HMM complexes (see below).

As an alternative approach to examine whether A3G in virion cores were indeed freely soluble or associated with other factors, cores purified by solubilization of whole virions (Figure 1C) were biochemically disassembled by exposure to a low pH “STE” buffer at 37 °C, as previously described [33]. This treatment resulted in release of p24-CA into the supernatant of a pelletable viral RNP complex consisting of IN, NC, and viral genomic RNA. Under these conditions, RT is more readily released from the RNP upon biochemical fractionation of the cores (Figure 3C and as previously described [34,35]). Analysis of A3G-containing virion cores revealed that IVAC A3G cofractionated with the viral RNP proteins (Figure 3C), suggesting a continued association with the viral genomic RNA and/or NC protein.

### Enzymatic Activity of A3G in the IVAC Is Negatively Regulated by RNA Binding

**Figure 4:**
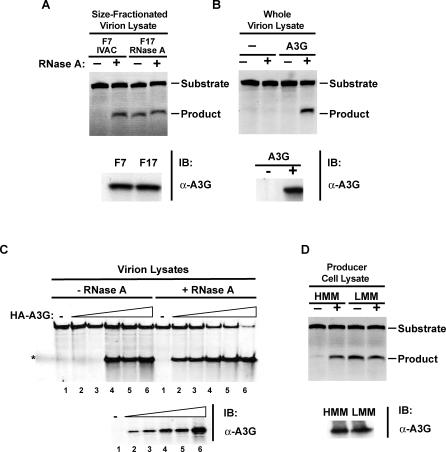
Intravirion A3G Enzymatic Activity Is Negatively Regulated by Binding to Genomic HIV RNA (A) HA-A3G was immunoprecipitated from IVAC fraction 7 (F7) of virion lysates (Figure 3A) or from a lower fraction, F17, generated by treatment of the virion lysates with RNase A (Figure 3B). Immunoprecipitates (IPs) were tested for enzymatic activity in an in vitro deoxycytidine deaminase assay with or without RNase A addition and contained equivalent amounts of HA-A3G as shown in the corresponding immunoblot. The generation of a shorter cleavage product from the input ssDNA substrate reveals A3G deoxycytidine deaminase activity. Data shown are representative of multiple experiments. (B) Lysates of virions containing or lacking A3G were assessed in the deaminase assay, with or without RNase A treatment. (C) Lysates of virions containing increasing amounts of HA-A3G (as shown in the corresponding immunoblot) were assessed in the deaminase assay, with or without RNase A treatment. The asterisk marks bleed-through of marker loaded to the left of the samples. The triangles represent the increasing dose of A3G relative to provirus and correspond to the sample numbers presented in Figure 1A. (A–C) All deaminase reactions were carried out in 50 mM Tris (pH 7.4) with (+) or without (−) RNase A, as indicated. (D) IPs of HMM or LMM HA-A3G from producer cell lysates were similarly assessed in the deaminase assay, with (+) or without (−) added RNase A. The IPs contained equivalent amounts of HA-A3G as shown in the corresponding immunoblot (IB).

Since virion-derived A3G ultimately exerts deoxycytidine deaminase activity during reverse transcription [1–6], we considered the possibility that A3G might remain enzymatically active even when bound to RNA in the IVAC. However, in an in vitro deoxycytidine deaminase assay, the IVAC HAA3G exhibited no detectable enzymatic activity (Figure 4A). Like the cellular HMM A3G complex (Figure 4D and [14]), deoxycytidine deaminase activity was readily detected when the virion HA-A3G immunoprecipitates were pretreated with RNase A before assessment (Figure 4A). Analysis of whole-virion lysate similarly showed that RNase A treatment was required for detection of A3G enzymatic activity in vitro (Figure 4B). These findings indicate that HA-A3G is incorporated into virions as an enzymatically latent large RNP complex. Of note, previous reports have observed readily detectable enzymatic activity from A3G-containing virions. We believe this may be due to the presence of additional, noncore LMM A3G packaged into the matrix space of virions upon A3G overexpression in cells (Figure 1C). When virions containing increasing amounts of A3G (Figure 1A) were tested for in vitro deaminase activity, substrate was readily deaminated by those virions which contained higher proportions of A3G to p24-CA than is normally packaged by infected CD4 T cells (Figure 4C). In addition, Yu et al. [6] reported that virion-derived A3G was enzymatically active in the absence of RNase treatment. However, in that study, virions were extracted in buffers containing EDTA [6], an agent that disrupts some RNA–protein complexes [36,37]. When we analyzed virion HA-A3G extracted in EDTA-containing buffers, we also detected deoxycytidine deaminase activity, suggesting that this treatment likely activated A3G by promoting its dissociation from inhibitory RNA(s) (Figure S3). Likewise, it has been observed that the addition of salts like magnesium that promote and stabilize RNA tertiary structure enhance the activity of recombinant A3G purified from insect cells while inducing a shift of A3G from large to intermediate-sized complexes [38]. Together these findings support the notion that RNA binding inhibits A3G enzymatic activity, likely by occluding the catalytic site.

### IVAC Contains HIV Genomic RNA

**Figure 5:**
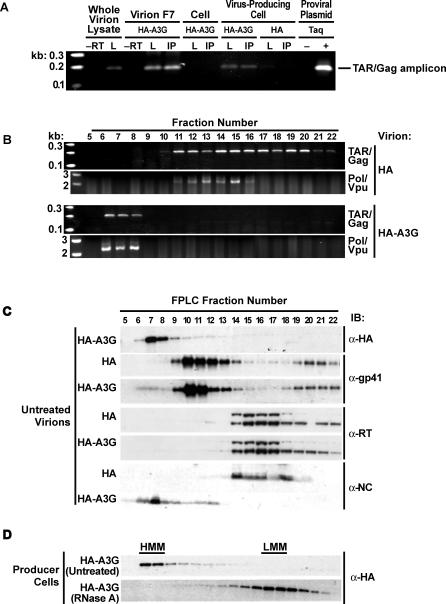
Virion-Incorporated HA-A3G Associates with Viral Genomic RNA (A) Viral genomic RNA, detected by RT-PCR, was detected in virions and virus-producing cells but not in lysates of uninfected cells. Genomic RNA was also detected in the IVAC derived from virions (fraction 7) and coimmunoprecipitated with HA-A3G from both virions and producer cell lysates. RT was performed using RNA derived from either whole lysates (L) or anti-HA immunoprecipitates (IP). Control reactions were performed in the absence of RT (–RT). Control PCRs were performed using proviral plasmid DNA, in the absence or presence of Taq, as indicated. (B) Viral genomic RNA, detected by RT-PCR, was assessed from size-fractionated virion lysates that lacked (HA) or contained HA-A3G. Amplicons generated probed across the TAR/Gag region or Pol/Vpu regions, as indicated. (C) Incorporation of HA-A3G into virions enhances the recruitment of NC into the IVAC. (D) HA-A3G from virus-producing cells is HMM and is converted to LMM form after RNase A treatment. “IB” indicates immunoblotting with the indicated antibody.

The reported recruitment of A3G into virions through viral RNA, the cofractionation of intravirion A3G with viral RNP proteins (Figures 1C and 3C), the assembly of A3G into a large RNase A–sensitive complex within virions (Figure 3A), and the RNase A–dependent in vitro enzymatic activity of intravirion A3G (Figure 4A and 4B) strongly suggested that HIV genomic RNA may be an important constituent, perhaps even a nucleating factor for IVAC assembly. To test this possibility, we immunoprecipitated A3G from IVAC fractions, purified the RNA, and subjected it to RT-PCR with primers that specifically amplify HIV genomic RNA. Genomic HIV RNA was readily detected in the IVAC as well as in A3G immunopre0cipitates prepared from virus-producing cells (Figure 5A).

Next, we compared the FPLC fractionation profile of HIV RNA derived from virions that contained or lacked HA-A3G. In the presence of HA-A3G, HIV RNA was detected in fractions that contained the IVAC, indicative of A3G-dependent assembly of large HIV RNA–protein complexes in virions (Figure 5B). In the absence of HA-A3G, viral RNA was detected in lower fractions 11 through 14. The fractions were determined to contain full-length genome by the production of PCR products using probes that amplify across various regions of the genome. However, these gel filtration experiments were associated with some RNA fragmentation, particularly in the absence of A3G (Figure 5B, TAR/Gag amplicons were observed in fractions 11 through 20). We suspect that such fragmentation also occurred in A3G-containing virions but that the RNA fragments continued to resolve in the IVAC fractions through persistent association with A3G.

Of note, virion NC, which also binds HIV RNA, was detected in the FPLC fractions that contained IVAC (Figure 5C). Conversely, RT, which is more readily released from HIV RNA upon biochemical manipulation/lysis of virions [34,35] (and Figure 3C), did not display a strong shift into the IVAC fractions in the presence of HA-A3G upon gel filtration. As expected, the non–RNP-associated gp41 viral protein resolved independently of A3G. In the absence of A3G, NC resolved in lower FPLC fractions, consistent with pools of protein that either have dissociated from the viral RNP or remain associated with RNA fragments (possibly caused by the gel filtration conditions, as discussed above). In both the absence [14] and presence of HIV gene expression (Figure 5D), cellular A3G resolves as HMM upon gel filtration.

### Inactive A3G in the IVAC Is Activated by Viral RNase H during Reverse Transcription

**Figure 6:**
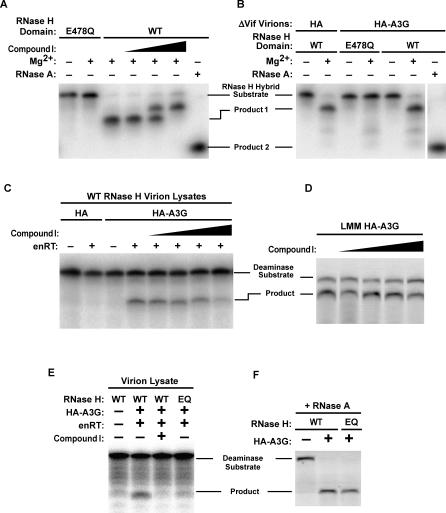
Enzymatically Inactive Virion-Incorporated HA-A3G Is Activated by Viral RNase H (A) Recombinant RTs containing either a WT or mutant (E478Q) RNase H catalytic domain were assessed for RNase H activity in vitro in the absence or presence of the RNase H inhibitor Compound I (final concentration of 1, 10, or 100 μM). The RNA of an RNA–DNA hybrid remains intact unless RNase H digests the RNA into a smaller cleavage product that is distinguishable from the more complete cleavage product generated by RNase A. WT RNase H cannot digest ssDNA or DNA of an RNA–DNA hybrid, or RNA–RNA hybrids (data not shown). RNase H assays were performed in RNase H buffer (50 mM Tris [pH 8.0], 60 mM KCl) with (+) or without (−) 5 mM MgCl_2_ or RNase A, as indicated. (B) Viruses bearing the RNase H E478Q mutation are compromised for in vitro RNase H activity. RNase H assays were performed in RNase H buffer with (+) or without (−) 5 mM MgCl_2_ or RNase A, as indicated. (C) Virion lysates were subjected to endogenous reverse transcription (enRT) conditions with or without Compound I (final concentration of 0.1, 1, 10, or 100 μM), and A3G activity in these samples assessed in the in vitro deoxycytidine deaminase assay. Deaminase assays were performed in RNase H buffer either supplemented (enRT:+) or not (enRT:−) with 4 mM MgCl_2_ and 1 mM dNTPs. (D) Compound I does not inhibit the intrinsic deoxycytidine deaminase activity of A3G. HA-A3G from RNase A–treated virion lysates was assessed for in vitro deaminase activity in the presence of increasing doses of Compound I (0.1, 1, 10, and 100 μM). Deaminase assay was performed in RNase H buffer supplemented with RNase A only. (E) Virions containing WT RNase H or the E478Q mutation in the RNase H catalytic domain were subjected to the enRT reaction followed by assessment of A3G enzymatic activity. Deaminase assays were performed in RNase H buffer either supplemented (enRT:+) or not (enRT:−) with 4 mM MgCl_2_ and 1 mM dNTPs. (F) WT and RNase H–compromised ΔVif virions containing WT or mutant RNase H displayed equivalent A3G activity when RNase A was added to the virion lysate. Deaminase assay was performed in RNase H buffer with (+) or without (−) RNase A, as indicated. All data are representative of multiple experiments.

Next our studies focused on how the latent deoxycytidine deaminase activity of A3G present in the IVAC is ultimately activated. As shown in Figure 4A and 4B, the simple addition of single-stranded DNA (ssDNA) substrate was insufficient for triggering its activity. In view of the effects of RNase A treatment in vitro, we were intrigued by the possibility that viral RNase H enzyme might play a role in the activation of A3G enzymatic activity. RNase H resides near the C-terminus of the large subunit of the p66-p51 RT heterodimer [39,40]. The DNA-dependent action of RNase H is required for commencement of second-strand synthesis and concomitantly generates the free, minus-strand ssDNA substrate that is targeted by A3G for deamination [2–6]. We hypothesized that viral RNase H action might not only generate the substrate for A3G-mediated deamination but also reverse the RNA-mediated inhibition of A3G deoxycytidine deaminase activity by degrading the genomic RNA bound to A3G. To examine this possibility, we established in vitro conditions that lead to RNase H activity and assessed the effects of active RNase H on A3G deaminase activity. Both recombinant purified RT and virion-derived RT cleaved an end-labeled RNA oligonucleotide from an RNA–DNA hybrid substrate (Figure 6A and 6B). Importantly, RNase H activity was magnesium dependent [41], was inhibited by a variety of small molecules including Compound I [42,43], and was compromised by specific mutations within its catalytic domain, for example, E478Q ([44] and Figure 6A and 6B). We used these various properties of RNase H and reagents to probe the potential involvement of RNase H as an activator of latent A3G deoxycytidine deaminase activity within virions.

First, we tested whether stimulation of endogenous reverse transcription in virion lysates by the addition of magnesium and deoxynucleotide triphosphates promoted activation of A3G enzymatic activity measured in the deaminase assay. Such treatment effectively induced readily detectable deoxycytidine deaminase activity, suggesting a link between reverse transcription and A3G activation (Figure 6C). Of note, the appearance of deoxycytidine deaminase activity was blocked in a dose-dependent manner by Compound I, an RNase H inhibitor. Importantly, Compound I did not impair the deoxycytidine deaminase activity of A3G induced by prior RNase A treatment (Figure 6D), supporting inhibition of RNase H as the cause of Compound I–mediated inhibition of A3G enzyme activation. Additionally, the introduction of a point mutation in the catalytic core of RNase H (E478Q), which compromised RNase H activity (Figure 6B), also impaired activation of A3G deoxycytidine deaminase activity under conditions permissive for endogenous reverse transcription (Figure 6E). HA-A3G was otherwise equally active upon RNase A treatment of viruses bearing either WT or compromised RNase H domains (Figure 6F). These findings demonstrate that, in addition to generating the substrate for A3G-mediated deamination, HIV-1 RNase H plays a central role in triggering the activity of the latent virion-associated A3G enzyme.

## Discussion

Our observation that A3G packages into HIV virion cores is not unexpected given that RNA and/or the NC region of Gag recruit the enzyme into the virus [15–22]. Further, A3G antiviral activity is ultimately manifested during reverse transcription and therefore proximity to reverse transcription complexes would be anticipated. Indeed, Khan et al. [18] reported core localization of A3G. However, it is clear from the virion fractionations that additional enzyme may gain access to the virion when A3G is overexpressed in virus-producing cells (Figure 1). This additional A3G is not specifically recruited into virion cores. It has been previously demonstrated that roughly twice as much Gag than that present in the virion core is incorporated into immature particles [30]. Based on the RNase A sensitivity of the interaction between Gag (NC) and A3G [16,18,19,22] and the ability of A3G to interact with highly divergent Gag proteins [21,23,45–47], it has been suggested that A3G may recognize an NC–RNA interface that promotes virion incorporation [23,47]. Alternatively, the conflicting reports regarding A3G virion recruitment by Gag/NC in an RNA-dependent [16,18,19,22] or RNA-independent [15,17,20,21] manner could be a consequence of the relative amounts of additional, non–core packaging occurring under the conditions of assay. The RNase-sensitivity of Gag interaction may be observable only at lower (endogenous-like levels) A3G concentrations where affinity is perhaps governed primarily by RNA interactions. At higher expression levels of A3G, a lower affinity but direct interaction with Gag independent of RNA may occur. Whether virion core–incorporated A3G, like NC, coats the viral RNA or whether A3G binding is restricted to certain regions [18] remains to be determined.

Differences in the absolute amount of A3G packaged into virions may also contribute to apparent disparities in prior studies of A3G. Within this study, for example, the additional packaging of A3G under conditions of A3G overexpression masked the RNase-dependent “activation” of virion A3G (Figure 4C), highlighting the importance of establishing conditions that closely recapitulate physiological levels of A3G incorporation into virions.

Several observations in this study support the notion that A3G incorporated into HIV virion cores is assembled into a large RNA–protein complex that we have termed the IVAC. First, A3G was incorporated into virion cores (Figure 1C), which contain viral RNP complexes consisting of viral genomic RNA, NC, IN, and Vpr. IVAC A3G both coimmunoprecipitated viral genomic RNA (Figure 5) and cofractionated with the virion core proteins (Figure 3C). Additionally, the shift in viral genomic RNA from lower to higher mass FPLC fractions upon HA-A3G expression supports the notion that RNA is critical for virion packaging of A3G and suggests its possible central role in nucleation of the IVAC. Importantly, although the resolving power of our fractionation is currently not able to differentiate the sizes of the cellular HMM A3G complexes and IVAC (all resolve at or near the void volume of the Superose 6 column), these complexes are not identical. For instance, the cellular HMM complexes form in the absence of viral genomic RNA in activated but uninfected CD4 T cells [14], while IVAC A3G interacts with HIV RNA (Figure 5).

Second, our preliminary results indicate that many of the protein components of the cellular HMM A3G complex [27,28,48] are not corecruited into HIV virions (unpublished data and [28]). Finally, the activation of IVAC A3G by in vitro endogenous reverse transcription (Figure 6) suggests that viral RNA inhibits IVAC A3G enzymatic activity unless removed by RNase H, a virally encoded enzyme that acts on the RNA component of RNA–DNA hybrids. Of note, the level of A3G activation obtained when endogenous reverse transcription is stimulated (Figure 6E) was consistently less robust than the level of enzymatic activity observed when the IVAC was treated with RNase A (Figure 6F). We suspect this finding reflects a more complete clearance of RNA from IVAC A3G by exogenously added RNase A than occurs with RNase H activation under conditions of endogenous reverse transcription. Alternatively, A3G incorporated into HIV virions may bind both HIV RNA and non-HIV RNA (for example, the tRNA-^Lys3^ primer); however, since only the viral RNA genome is reverse transcribed, thereby forming a substrate for RNase H activity, only viral RNA-bound A3G may become “activated” during reverse transcription.

The pulse-chase radiolabeling studies of A3G in cells revealed that newly synthesized, LMM A3G is rapidly (within 30 min) recruited into cellular HMM complexes (Figure 2A) and that proviral gene expression has little, if any, effect on A3G assembly into HMM RNA–protein complexes (Figure 2B). Extension of the pulse radiolabeling time did not impede assembly but masked detection of the rapid assembly of newly synthesized LMM A3G into HMM complexes, in both the absence (Figure 2C) and the presence (Figure 2D) of viral gene expression. Notably, these complexes appear to be stable for at least several hours (Figure S2B).

The assembly of intravirion A3G into a large RNP complex could result from recruitment of any of the cellular HMM A3G complexes (Staufen RNA transporting granules, Ro/La RNPs) into virions or by HIV RNA extraction of A3G from cellular HMM A3G complexes. Alternatively, newly synthesized A3G not yet assembled into fully mature cellular HMM complexes could bind to HIV RNA, which in turn targets the enzyme for encapsidation into HIVΔVif virions. As noted, A3G assembled into HMM A3G complexes or A3G assembled with HIV RNA and core proteins sieve near the void volume of the Superose 6 columns and thus these different types of complexes cannot be distinguished by FPLC. To investigate whether newly synthesized A3G or older, “mature” A3G already assembled into HMM complexes is recruited into HIV virions, virus-producing cells were subjected to pulse-chase radiolabeling studies. In each of three experiments, we observed the appearance of a peak of radiolabeled A3G in virions at one discrete time point, occurring between 0.5 h and 1 h after the pulse (Figure 2E, middle panels). Radiolabeled A3G incorporation into virions decreased dramatically after this peak, despite the persistence of substantial pools of radiolabeled A3G in the producer cells from which the virions were derived (Figure 2E, top panel). The loss of radiolabeled A3G in virions after this peak also could not be explained by a sharp decline of radiolabeled A3G in the producer cells (Figure S2C). These findings suggest that once A3G assembles into the cellular HMM A3G complexes [27,28,48], it may no longer serve as a major reservoir of enzyme for virion encapsidation. Pulse radiolabeling of A3G before the peak of viral Gag expression and extension of the collection times up to 9 h after the pulse further confirmed that mature HMM cellular A3G does not form a major pool of enzyme for incorporation into HIVΔVif virions (Figure S2E).

Instead, it appears that newly synthesized A3G is preferentially recruited into HIV virions within 1.5 h after synthesis (Figure 2E, middle panels). Interestingly, the appearance of radiolabeled HA-A3G in virions appeared to be slightly delayed since samples collected (1) immediately after the pulse and (2) in the first 30-min chase contained relatively little radiolabeled A3G compared to virions collected over the second 30-min chase (Figure 2E, 1-h collection time point). This is clearly within the time required for assembly of newly synthesized A3G into cellular HMM complexes (within 30 min). Although virions were budded during the 30-min pulse, newly synthesized Gag did not yet contribute to these virions until the first 30-min chase period (Figure 2E, lower panels). Thus, the recruitment of newly synthesized A3G into virions may be intimately tied to the synthesis and assembly of the viral genome and/or Gag (NC) and the budding of these new virions. Indeed, A3G assembles with viral RNA in producer cells (Figure 5 and [28]) and maintains this association within virion cores (Figures 3 and 5). Since newly synthesized A3G assembles into HMM complexes in cells within 30 min in the presence or absence of HIV RNA (Figure 2A–2D), we cannot determine in these experiments whether the newly synthesized A3G (less than 1.5 h old) recruited into virions represents A3G newly assembled into any one specific cellular complex, a viral-specific HMM complex, or a combination of cellular and viral complexes. However, several other observations in conjunction with these pulse-chase data strongly support a model in which newly synthesized cellular A3G not yet fully assembled into cellular HMM complexes forms the major pool for recruitment into HIVΔVif virions. First, A3G incorporation into virions is mediated by assembly with viral determinants for encapsidation, including the viral RNA genome and/or Gag [15–22], and virion-incorporated A3G ultimately forms a large RNP complex (IVAC) with viral RNA, NC, and IN (Figures 3 and 5). Thus, virion-bound A3G forms a complex distinct from the cellular HMM complexes, at the very least distinguished by the presence of the viral encapsidation determinants (viral RNA genome and/or Gag). Second, since none of the cellular cofactors identified in the cellular HMM A3G complexes are corecruited with A3G into virions in an A3G-specific manner ([28] and unpublished data), viral determinants for A3G virion incorporation would have to extract A3G out of mature multisubunit complexes if they do serve as a reservoir for virion incorporation. If such a mechanism is employed, it is difficult to explain why A3G incorporation into virions is not also readily detected at much later time points in the pulse-chase radiolabeling studies. Finally, overexpression of A3G in cells leads to packaging of additional amounts of A3G into virions that localize outside of the virion core (Figure 3C), and this form of A3G is enzymatically active in vitro in the absence of addition of RNase A (Figure 4C). Thus, this additional extra-core A3G appears to be the LMM monomer/dimer that forms upon RNase A treatment [14] and could possibly arise from (1) newly synthesized LMM A3G not yet assembled into HMM complexes or (2) LMM A3G not assembled into HMM complexes due to saturation of cellular cofactors upon A3G overexpression.

We thus favor a model in which, upon translation, newly synthesized LMM A3G assembles with viral RNA and protein factors to gain access to newly assembling virions (and, in so doing, forms an IVAC-like complex). Since viral genomic RNA is subject to cellular processing that may be common to RNA that nucleates the cellular HMM complexes, a subset of common cellular RNA-binding factors can be predicted to be found in both the cellular HMM complexes [27,28,48] and viral RNP complexes. For example, RNA helicase A, a component of the Staufen-containing HMM A3G complex, has been reported to be packaged into virions [49], but its incorporation occurs independently of A3G and is unaffected by A3G virion incorporation (unpublished data). However, we cannot completely exclude the possibility that A3G is recruited into virions by viral cofactors from very recently assembled HMM complex(es), as recently suggested [28]. However, because we do not observe virion incorporation from older HMM A3G complexes, we do not favor such a model. One limitation of the pulse-chase studies is that we cannot calculate the percentage of radiolabeled (newly synthesized) to unlabeled (mature) A3G that is in virions at any of the given collection times.

The recruitment of A3G into HIV virions is ultimately detrimental to the virus, underscoring the essential function of the HIV Vif protein in blocking encapsidation of the deaminase. The principal mechanism by which Vif abrogates antiviral A3G activity is believed to involve proteasome-mediated degradation of A3G, most of which is resident in HMM A3G complexes. The observation that newly translated A3G (less than 1.5 h old) is preferentially recruited into virions (Figure 2B) implies that Vif must also effectively target this newly synthesized pool of cellular A3G. Recently, it has been reported that more Vif binds to A3G in the presence of RNase that in its absence [28], suggesting that LMM A3G unbound to RNA may be a good target for Vif. Our prior studies have shown that Vif expression promotes polyubiquitinylation of A3G that resolves as HMM [14]. Whether Vif activity leads to A3G ubiquitylation before, during, or after the assembly of this enzyme into cellular HMM complexes remains to be determined. Similarly, it remains to be determined whether ubiquitylated A3G resolving into HMM fractions upon FPLC represents the modification of A3G in the recently identified cellular complexes [27,28,48] or a separate complex of A3G, Vif, Cul5, SCF, and the proteasome. Perhaps Vif targets ribosome-associated A3G, thus destroying newly synthesized A3G and removing the key pool of enzyme that is selectively incorporated into virions. Such a scenario is consistent with the observation that Vif partially inhibits the synthesis of A3G [7,50]. Alternatively, Vif could target A3G bound for virion incorporation by targeting viral RNA–associated A3G. Indeed, Vif has been reported to interact with viral genomic RNA, suggesting a mechanism by which it might preferentially target virion-bound A3G [51–53]. Similarly, the reported association of Vif with the plasma membrane [54,55], the site of virion assembly, could localize this viral protein in proximity to A3G undergoing active encapsidation. While Vif expression ultimately depletes cells of all A3G, others have suggested that such global degradation of the enzyme may not be strictly required for Vif to exert its countereffects on A3G [56,57]. Regardless, our findings suggest that an important target for Vif is the newly synthesized pool of A3G, rather than A3G already assembled into cellular HMM complexes.

Because A3G can hypermutate the nascent minus-strand DNA of HIV, we found it surprising that intravirion A3G is inactive in in vitro deoxycytidine deaminase assays. Indeed, we observed that the binding of HIV RNA to A3G within virions prevented ssDNA binding and/or occluded the A3G catalytic site(s). As shown in Figure 4A, the addition of free ssDNA substrate to virion A3G proved insufficient to compete RNA binding and/or access the catalytic pocket(s). Rather, the inhibitory RNA had to be removed before A3G enzymatic activation was observed. In view of the emerging findings that A3G can also exert antiviral activity independent of deoxycytidine deamination [14,46,58–62], we propose that virion A3G may employ two mechanisms acting sequentially to produce its full antiviral effect. First, the enzymatically latent form of A3G bound to HIV RNA may impair the generation of minus-strand DNA by physically blocking the movement of RT on its viral RNA template. Indeed, short interfering RNA–mediated knockdown of endogenous A3G in resting CD4 T cells enhances the synthesis of late reverse transcription products [14] and the generation of both early and late reverse transcription products is reduced by the presence of A3G in virions [63]. However, because this inhibition is incomplete, minus-strand viral DNA is occasionally generated, setting the stage for the second, enzyme-dependent antiviral action of A3G. During reverse transcription, we now show that RNase H degrades the viral RNA that impairs A3G activity, allowing the enzyme to extensively deaminate the minus-strand DNA. Perhaps incomplete inhibition of reverse transcription by A3G is caused by the occasional ability of RT displace A3G off the RNA template. Although this could result in the generation of enzymatically active A3G, A3G may also be able to rebind the RNA–DNA duplex, reestablishing the inactivated state and dependence upon RNase H for enzymatic activation. Indeed, the lack of A3G activity induced by reverse transcription but under conditions where RNase H activity is inhibited (Figure 6E) suggests that if A3G is displaced by RT, it rapidly rebinds inhibitory nucleic acid. These events of initial inhibition and subsequent activation of A3G enzymatic activity by various components of the virus highlight an unexpectedly complex but interesting interplay between HIV and its cellular host.

Such a dual strategy for A3G inhibition of retroviral replication could account for its potent antiviral activity and explain reports of both enzyme-dependent and -independent antiviral activities. This model could also explain the conflicting results concerning the ability of A3G to inhibit the replication of hepatitis B virus. In some cells, A3G acts independently of deoxycytidine deamination [46,58], while in others, prominent DNA mutation is evident [64–66]. Cell-type differences in the relative effectiveness of these two sequential antiviral actions of A3G could underlie these findings.

## Materials and Methods

### Cells and viruses.

The 293T cells were maintained in DMEM supplemented with 10% FBS (Gemini Bio-Products, http://www.gembio.com). H9 cells were maintained in RPMI supplemented with 10% FBS. Primary CD4 T cells were isolated from fresh human peripheral blood mononuclear cells on CD4 magnetic microbeads (Miltenyi Biotec, http://www.miltenyibiotec.com). The isolated CD4 T cells were then activated by 36-h treatment with PHA (5 μg/ml) followed by 36-h IL-2 treatment (20 U/ml; Roche, http://www.roche.com) in RPMI supplemented with 10% FBS, 100 μg/ml streptomycin, and 100 U/ml penicillin. Virions were generated by calcium phosphate–mediated cotransfection of subconfluent 293T in T175 flasks with a proviral plasmid (60 μg), pCMV4-HA-A3G vector (0 to 20 μg), and/or pCMV4-HA (0 to 20 μg). The medium was changed after 16 h, and the supernatant and cells collected were collected after 48 h. The virus-containing supernatant was clarified by low-speed centrifugation, filtered through a 0.22-μm membrane, and sedimented by ultracentrifugation over a 2-ml cushion of 8.4% iodixanol at 20,000 rpm using an SW28 rotor (Beckman Coulter, http://www.beckmancoulter.com) for 2 h at 4 °C. The virus-containing pellet was resuspended in 1 ml of PBS, DNase-treated (RNase-free; Roche), underlaid with a 100-μl cushion of 8.4% iodixanol and ultracentrifuged at 20,000 rpm in an HFA 22.1 rotor (Heraeus, http://www.thermo.com) for 1 h at 4 °C. Unless otherwise indicated, 0.1 U of RNase A inhibitor (RNaseOUT; Invitrogen, http://www.invitrogen.com) was added to virion pellets, which were then immediately lysed or flash-frozen on liquid nitrogen and stored at −80 °C until lysis. The addition of RNaseOUT had no effect on the intrinsic activity of HA-A3G (Figure S4). Cells were washed with PBS, and the pellet was either immediately lysed or flash-frozen on liquid nitrogen and stored at −80 °C until use. To generate VSV-G–pseudotyped ΔVif virions, 293T cells were cotransfected with expression vectors for the ΔVif provirus and the envelope of VSV-G. At 48 h after transfection, supernatants were cleared by low-speed centrifugation and filtration as described above and then used directly on fresh H9 or primary CD4 T cells. The T cells were spinoculated with the pseudotyped virion-containing supernatant as previously described [67]. Briefly, 0.4 × 10^6^ cells/well of a 48-well plate were centrifuged at low speed for 2 h at room temperature with VSV-G–pseudotyped viruses. Cells were then washed five times with cold medium and returned to complete media for an additional 40 h. Supernatants and cells were then collected and processed as described above for the transfected 293T cells.

### Plasmids.

The proviral clone of pNL4–3ΔVif used to generate the HIV-1ΔVif virions has been previously described [68,69]. pNL4–3ΔVifH^−^(E478Q) contains a point mutation in the catalytic site of the RNase H domain of RT that compromises RNase H activity. This plasmid was generated by first subcloning the SpeI-EcoR1 Pol-containing restriction fragment of pNL4–3ΔVif into pEF1A. The mutagenesis primer 5′–ACAACAAATCAGAAGACTCAGTTACAAGCAATTCATCTAGC–3′ and its complement (Operon, http://www.operon.eu.com) were used to generate the E478Q mutation in the subclone using the QuikChange site-directed mutagenesis kit (Stratagene, http://www.stratagene.com). The mutation was confirmed by DNA sequencing. The pol region in the subclone was then recloned back into pNL4–3ΔVif. The introduction of the E478Q mutation into NL4–3ΔVifH^–^(E478Q) was confirmed by sequencing. pCMV4-HA and pCMV4-HA-A3G [7] expression vectors were cotransfected with pNL4–3ΔVif to generate HIV-1ΔVif virions lacking or containing HA-tagged A3G.

### Virion fractionations.

Virion cores were obtained using a previously published method [29]. Briefly, virion pellets were resuspended in MOPS Buffer I (200 mM NaCl, 100 mM MOPS [pH 7.0]) and Triton X-100 added to a final concentration of 0.5% for 2 min at room temperature. The cores were then pelleted from the solubilized enveloped by spinning the samples at 14,000*g* for 8 min at 4 °C. The core pellets were then washed twice with MOPS buffer II (100 mM NaCl, 50 mM MOPS [pH 7.0]). The cores were then either analyzed by immunoblotting or further fractionated to remove the p24-CA shell, as previously described [33]. Briefly, cores were resuspended in STE buffer (10 mM Tris [pH 6.7], 1 M NaCl, 0.5 mM EDTA), incubated at 37 °C for 4 h and subsequently centrifuged at 14,000*g* to pellet the RNP complex.

### FPLC analyses.

Virions present in the supernatants of 293T cells were transiently transfected with HIV proviral plasmids, and the cells themselves were lysed in ice-cold lysis buffer (50 mM HEPES [pH 7.4], 125 mM NaCl, 0.2% NP-40, and 1× EDTA-free protease inhibitor cocktail [Calbiochem/EMD Biosciences, http://www.emdbiosciences.com]). Lysates were clarified by sedimentation, quantified with a protein assay (Bio-Rad, http://www.bio-rad.com), and applied to a calibrated Superose 6 HR 10/30 gel filtration column run by an FPLC apparatus (AKTA; Amersham Biosciences, http://www.amersham.com). One column-volume (24 ml) using FPLC running buffer (50 mM HEPES [pH 7.4], 125 mM NaCl, 0.1% NP-40, 1 mM dithiothreitol, and 10% glycerol) was collected in 1-ml aliquots. Equal volumes of collected fractions were either directly run on SDS-PAGE gels or concentrated with YM-3 Microcon filters with a cutoff of 3,000 Da (Millipore, http://www.millipore.com) before running on SDS-PAGE after normalization for resultant concentrate volume. The size-separated proteins were then transferred to nitrocellulose membranes and immunoblotted. To test nuclease sensitivity, the lysates were pretreated with 50 μg/ml RNase A (DNase-free; Roche) and/or 20 to 200 U/ml DNase (RNase-free; Roche) for 1 h at 37 °C before gel filtration.

### Antibodies.

Polyclonal antibodies against A3G [7] and Vpr [70] have been previously described. Through the National Institutes of Health AIDS Research and Reference Reagent Program, HIV-1 RT monoclonal antibody (8C4) was obtained from Dr. Dag E. Helland, polyclonal antiserum to HIV-1 IN (757) was obtained from Dr. Duane Grandgenett, and HIV-1 gp41 human antibody (No. 50–69) was obtained from Dr. Susan Zolla-Pazner. Anti–NC-p7 antibody was generously provided by Dr. Robert J. Gorelick (National Cancer Institute, Frederick, Maryland, United States). Mouse monoclonal anti-p24 Gag ascites was generously provided by Beckman Coulter. Other antibodies used include polyclonal anti-HA antibody Y11, monoclonal anti–14-3-3γ antibody C-16, monoclonal anti-CD45 antibody 2D-1, and polyclonal anti-GFP antibody (FL) (all Santa Cruz Biotechnology, http://www.scbt.com) and monoclonal anti-HA antibody HA.11 unlinked or linked to beads (Covance, http://www.covance.com).

Immunoblot analysis of proteins was performed using horseradish-linked secondary antibodies followed by ECL detection (Pierce Biotechnology, http://www.piercenet.com). In Figure 1, A3G and p24-CA were detected and quantified by using fluorescently linked secondary antibodies (LI-COR Biosciences, http://www.licor.com). Blotted proteins were then detected and quantified using the Odyssey infrared imaging system and software (LI-COR).

### Pulse-chase radiolabeling experiments.

Four plates of 293T cells were transfected with pCMV4-A3G-HA alone or with pNL4–3ΔVif. After 36 h, the cells were rinsed once and incubated for 1 h with pulse-radiolabeling medium (DMEM without methionine and cysteine; GIBCO, http://www.invitrogen.com) plus 10% dialysed FBS). The cells were pulse labeled for 10 min with 500 μCi/ml EasyTag XPRESS ^35^S Protein Labeling Mix (Perkin Elmer, http://www.perkinelmer.com) containing radiolabeled methionine and cysteine in fresh pulse-radiolabeling medium. At the end of the pulse-radiolabeling period, the radiolabel was removed and one plate of cells harvested. The remaining radiolabeled samples were incubated with chase medium (DMEM supplemented with 10% FBS, 4.02 mM methionine [20×], and 3 mM cysteine [15×]). Cells were harvested following incubation for 0.5, 1, or 2 h. Cells pellets were lysed in ELB lysis buffer. Each lysate was size-fractionated on gel filtration columns packed with Sepharose CL-6B beads, which crudely separate HMM from LMM proteins (Figure S1). For each sample, ten fractions of 300 μl each were collected, and equal volumes of each fraction were immunoprecipitated with anti-HA antibody. The immunoprecipitates were run on SDS-PAGE, and the signal was detected by autoradiography. The signal from each radiolabeled A3G-HA band was quantitated using Scion Image for Windows software (Version 1.62; Scion Corporation, http://www.scioncorp.com) and divided by the sum of the total signal, in order to assign a relative percent density versus the fraction number for every chase time point sample.

For the pulse-chase analysis of virus-producing cells, 293T cells were cotransfected with pNL4–3ΔVif, pCMV4-HA-A3G, and pEGFP-C1 to generate HA-A3G–containing HIV-1ΔVif virions. After 48 h, the medium was changed, and the cells were rinsed and incubated for 1 h with pulse-radiolabeling medium as described above. The cells were then pulse-radiolabeled for 30 min with 125 μCi/ml EasyTag XPRESS ^35^S Protein Labeling Mix (Perkin Elmer) in fresh pulse-radiolabeling medium. At the end of the pulse-radiolabeling period, the radiolabel was removed. Supernatant from the initial pulse-labeled samples (*t* = 0, pulse) was harvested, and radiolabeled cells were incubated with chase medium (DMEM supplemented with 10% FBS, 4.02 mM methionine [20×], and 3 mM cysteine [15×]) for 0.5 h. Again, supernatant was collected (*t* = 0.5 h), and the cells were incubated with chase medium for a further 30 min to generate the *t* = 1 h sample. The process was repeated twice more with an incubation of 1 h and 2 h to generate the *t* = 2 h and *t =* 4 h samples. At all time points, a fraction of radiolabeled cells were also collected, washed with PBS, pelleted by centrifugation, flash-frozen on liquid nitrogen, and stored at −80 °C. The virus-containing supernatants were filtered through a 0.22-μm membrane, and virions were sedimented by ultracentrifugation over a 2-ml cushion of 8.4% iodixanol at 20,000 rpm in an SW28 rotor (Beckman) at 4 °C. The pellets were resuspended in 1 ml of PBS, underlaid with a 100-μl cushion of 8.4% iodixanol, and ultracentrifuged at 20,000 rpm in an HFA 22.1 rotor (Hereaus) for 1 h at 4 °C. The resultant virion pellets were flash-frozen on liquid nitrogen and stored at −80 °C.

After virion and cell pellets had been obtained for all time points, the samples were lysed in the lysis buffer described above. Lysates were clarified by sedimentation and quantified with a protein assay (Bio-Rad), and immunoprecipitations were set up at equal protein concentration/volume in the presence of monoclonal anti-p24 ascites or monoclonal anti-HA antibody and incubated for 2 h at 4 °C. The immunoprecipitates were washed once with lysis buffer and subjected to SDS-PAGE. The proteins were transferred to nitrocellulose and immunoblotted for GFP or HA with polyclonal antibodies or for p24 with monoclonal antibody.

GFP, HA-A3G, and p24-CA identified by immunoblotting were excised from the membranes and subjected to scintillation analysis. Bands were first identified by immunoblotting since Gag and HA-A3G coimmunoprecipitate with each other [15,17,20,21] and are close in size. Scintillation counts were normalized to the amount of immunoprecipitated material assessed, determined with ImageJ (http://rsb.info.nih.gov/ij). The normalized counts were divided by the sum of the total counts to assign a relative percent density for every sample. No GFP was detected in virions (unpublished data).

In an alternate approach (Figure S2E), 293T cells were first transfected with HA-A3G expression vector DNA using Fugene (Roche) followed by infection of these cells with VSV-G-pseudotyped NL4–3ΔVif for 12 h. The cells were then pulse-radiolabeled with 125 μCi/ml EasyTag, as described above. Also as described above, after the pulse, cells were chased with cold medium and cells and virions were harvested at 1, 3, 5, and 9 h after the pulse-radiolabeling period. In these experiments, samples were subjected to denaturing lysis (50 mM Tris [pH 7.5], 1% SDS, 5 mM dithiothreitol) followed by anti-HA or anti-p24 immunoprecipitations (50 mM Tris [pH 7.5], 250 mM NaCl, 5 mM EDTA, 0.5% NP-40) and immunoblotting or PhosphorImaging (Bio-Rad), as indicated.

### In vitro deoxycytidine deaminase assays.

Samples for analysis were either (1) whole virion lysates or (2) FPLC fractions from cell or virion lysates. FPLC fractions were immunoprecipitated with monoclonal anti-HA antibody to concentrate HA-A3G. In all cases, the amount of HA-A3G in the input samples was confirmed by immunoblotting before analysis. DNA oligonucleotides (5′-ATTATTATTATTC*C* *C*ATTTATTTATTTATTTATGGTGTTTGGTGTGGTTG-3′) containing target sites for A3G deamination [italicized] were labeled at the 5′ end with [^32^P]ATP using T4 polynucleotide kinase (New England Biolabs, http://www.neb.com) or with an FITC fluorophore (Operon). Labeled oligonucleotides and input samples were incubated in 20 μl of 50 mM Tris buffer (pH 7.4), with or without RNase A (1 μg) at 37 °C for 3 h unless otherwise indicated. For incubations under conditions stimulating endogenous reverse transcription, KCl (final concentration, 60 mM), MgCl_2_ (final concentration, 4 mM), and dNTPs (final concentration, 1 mM) were added. The RNase H inhibitor Compound I, generously provided by Dr. Daria Hazuda (Merck), was used at a final concentration of 0.1, 1, 10, or 100 μM. To terminate the reactions and purify the labeled oligonucleotides, the reactions were subjected to G-25 Mini Quick Spin Columns (Roche). Any uracil bases generated by A3G were converted to abasic sites by treatment of the purified oligonucleotides with 1 U of uracil DNA glycosylase (New England Biolabs) for 30 min at 37 °C. After 10 min of heat inactivation at 95 °C, the reactions were subjected to alkaline hydrolysis by the addition of NaOH (final concentration, 0.2 M) for 10 min at 95 °C. Cleavage products were resolved on 15% PAGE TBE-urea gels (Bio-Rad) and visualized with a Personal FX Imager (Bio-Rad), for radiography or fluorescence.

### RNase H assays.

Samples for analysis were either virion lysates or recombinant protein. Recombinant HIV-1 RT, WT or E478Q, was generously provided by Dr. Matthias Gotte (McGill University) and used at a final concentration of 1 nM. Test substrates included ssDNA, ssRNA, RNA–RNA hybrid, and DNA–RNA hybrid. Unmodified 18-mer PAGE-purified complementary DNA and RNA oligonucleotides were from Operon and are based on the oligonucleotides 18-DAB-DNA and 18-FAM-RNA described by Shaw-Reid et al. [42]. In addition, an unmodified RNA oligonucleotide complementary to 18-FAM-RNA was used to generate the RNA–RNA hybrid (Operon). All oligonucleotides were end-labeled with [^32^P]ATP using polynucleotide kinase (New England Biolabs). Hybrids were formed by annealing hot oligonucleotide to cold complementary oligonucleotide. Samples were incubated in 20 μl of RNase H buffer (50 mM Tris-Cl [pH 8.0], 60 mM KCl) either with or without 5 mM MgCl_2_, as indicated, for 10 min at 37 °C. Compound I was added to a final concentration of 0.1, 1, 10, or 100 μM. Radiolabeled substrate (single-stranded or hybrid) was added to a final concentration of 100 nM, and the reactions were allowed to continue at 37 °C for 30 min. After the addition of loading dye to stop the reactions, the cleavage products were resolved on 20% PAGE-TBE-urea gels and were visualized with a Personal FX PhosphorImager (Bio-Rad).

### RT-PCR.

FPLC samples and immunoprecipitates for RNA analysis were first treated with 20 U of DNase (RNase-free; Roche) at 37 °C. The RNA was then extracted with the QiaAmp RNA purification kit (Qiagen) according to the manufacturer's instructions. Viral genomic RNA was detected by reverse transcription with a primer complementary to the gag region of HIV-1 (5′-TGCTATGTCACTTCCCCTTGG-3′, generously provided by Jerry Kropp [Gladstone Institute of Virology and Immunology]) followed by PCR using primers complementary to the R (F496; nucleotides 496–517) and U5 (R573; nucleotides 552–573) or U5 (F592; nucleotides 592–613) and PBS (R666; nucleotides 645–666) regions of HIV-1. All these primers have been described [14]. In addition, reverse transcription was also performed using an antisense primer complementary to the Vpu region (5′-TCATTGCCACTGTCTTCTGCTCT-3′) followed by PCR using the Vpr primer and a primer complementary to Pol (5′-GTAATATGGGGAAAGACTCCT-3′).

## Supporting Information

Figure S1 Analysis of the Purity of the Virions Generated (A) The 293T cells expressing ΔVif provirus with (+) or without (−) HA-A3G, and virions derived from these cells, were assessed by immunoblotting (IB) for an abundant cellular factor 14-3-3γ that is not incorporated into virions [71]. This factor was readily detected in the producer cell lysates but not in the virion lysates.
(B) Activated primary CD4 T cells derived from peripheral blood of two donors were spinoculated with VSV-G–pseudotyped NL4–3ΔVif. Both the infected cells and virions derived thereof were assessed by immunoblotting (IB) for the presence of CD45 (molecular weight 180–220 kDa), which is highly expressed on miscrovesicles but excluded from virions [72].
(452 KB AI).

Figure S2 Additional Pulse-Chase Data (A) Gel filtration of cell lysates by mini Sepharose CL-6B columns distinguishes HMM A3G from LMM A3G. The 293T cells transiently expressing A3G-HA or HA-A3G were lysed and subjected to CL-6B size-fractionation. The ten fractions collected were assessed by immunoblotting (IB) to determine the resolution of A3G and monomeric 55-kDa α-tubulin. Shown are eight representative gel filtrations. HMM A3G, which peaks in fractions 4 and 5, is readily distinguishable from LMM α-tubulin, which peaks in fractions 6 and 7.
(B) Pulse labeling for 30 min resolves newly synthesized A3G as HMM within the 30-min pulse period and persists as HMM for at least 3 h. Chase lysates were similarly subjected to gel filtration as in (A). Shown are the autoradiograms of the anti-HA immunoprecipitates from each fraction after gel filtration.
(C) Normalization of radiolabeled HA-A3G incorporated into virions (as originally plotted in Figure 2E, middle panel), by the amount of radioactive HA-A3G available in the producer cell lysates (Figure 2E, top panel). Plotted is the percent radioactive density of thus-normalized A3G for any given time point relative to the total radioactive density of all the time points. P, pulse.
(D) The decline in radiolabel content over the chase is similar for HA-A3G in the absence or presence of ΔVif proviral gene expression. The 293T cells in a six-well plate were transfected with HA-A3G and GFP plasmids but in the absence of provirus, in parallel with the cells in Experiment 1 of Figure 2E. The pulse chase was performed as in Figure 2E, in parallel with the provirus-expressing samples. Plotted is the percent density of radiolabeled HA-A3G relative to the whole sample.
(E) Extension of the chase time reveals that even 9-h-old HMM A3G is not recruited into virions. The 293T cells transfected with HA-A3G were infected with VSV-G–pseudotyped HIVΔVif, pulse labeled, and then chased with cold medium for the following 9 h. HA-A3G and p24-CA were immunoprecipitated and subjected to either immunoblotting (IB) with antibodies to either A3G or p24-CA or PhosphorImager analysis, as indicated. P, pulse.
(4.9 MB AI).

Figure S3 Treatment of Virion Lysates with EDTA Activates A3G Enzymatic Activity Virions bearing a WT or mutant RNase H domain were lysed in lysis buffer supplemented with 10 mM EDTA and assessed in the deoxycytidine deaminase assay. Virions contained or lacked HA-A3G as indicated.
(964 KB AI).

Figure S4 A3G Enzymatic Activity Is Unaffected by RNaseOUT HA-A3G from RNase A–treated virion lysates was assessed in the in vitro deoxycytidine deaminase assay with increasing doses of RNaseOUT (0.2, 4, and 40 U, as indicated by the slope of the triangle) to assess whether the inhibitor affects intrinsic A3G enzymatic activity.
(1.4 MB AI).

## Abbreviations

A3G: APOBEC3G (apolipoprotein B mRNA-editing enzyme, catalytic polypeptide-like)
CA: Capsid
FPLC: fast protein liquid chromatography
HMM: high-molecular-mass
IN: integrase
IVAC: intravirion A3G complex
LMM: low-molecular-mass
NC: nucleocapsid
RNP: ribonucleoprotein
RT: reverse transcriptase
ssDNA: single-stranded DNA
WT: wild-type

## References

[ppat-0030015-b001] Sheehy AM, Gaddis NC, Choi JD, Malim MH (2002). Isolation of a human gene that inhibits HIV-1 infection and is suppressed by the viral Vif protein. Nature.

[ppat-0030015-b002] Harris RS, Bishop KN, Sheehy AM, Craig HM, Petersen-Mahrt SK (2003). DNA deamination mediates innate immunity to retroviral infection. Cell.

[ppat-0030015-b003] Mangeat B, Turelli P, Caron G, Friedli M, Perrin L (2003). Broad antiretroviral defence by human APOBEC3G through lethal editing of nascent reverse transcripts. Nature.

[ppat-0030015-b004] Lecossier D, Bouchonnet F, Clavel F, Hance AJ (2003). Hypermutation of HIV-1 DNA in the absence of the Vif protein. Science.

[ppat-0030015-b005] Zhang H, Yang B, Pomerantz RJ, Zhang C, Arunachalam SC (2003). The cytidine deaminase CEM15 induces hypermutation in newly synthesized HIV-1 DNA. Nature.

[ppat-0030015-b006] Yu Q, Konig R, Pillai S, Chiles K, Kearney M (2004). Single-strand specificity of APOBEC3G accounts for minus-strand deamination of the HIV genome. Nat Struct Mol Biol.

[ppat-0030015-b007] Stopak K, de Noronha C, Yonemoto W, Greene WC (2003). HIV-1 Vif blocks the antiviral activity of APOBEC3G by impairing both its translation and intracellular stability. Mol Cell.

[ppat-0030015-b008] Marin M, Rose KM, Kozak SL, Kabat D (2003). HIV-1 Vif protein binds the editing enzyme APOBEC3G and induces its degradation. Nat Med.

[ppat-0030015-b009] Sheehy AM, Gaddis NC, Malim MH (2003). The antiretroviral enzyme APOBEC3G is degraded by the proteasome in response to HIV-1 Vif. Nat Med.

[ppat-0030015-b010] Conticello SG, Harris RS, Neuberger MS (2003). The Vif protein of HIV triggers degradation of the human antiretroviral DNA deaminase APOBEC3G. Curr Biol.

[ppat-0030015-b011] Mehle A, Strack B, Ancuta P, Zhang C, McPike M (2004). Vif overcomes the innate antiviral activity of APOBEC3G by promoting its degradation in the ubiquitin-proteasome pathway. J Biol Chem.

[ppat-0030015-b012] Yu X, Yu Y, Liu B, Luo K, Kong W (2003). Induction of APOBEC3G ubiquitination and degradation by an HIV-1 Vif-Cul5-SCF complex. Science.

[ppat-0030015-b013] Mariani R, Chen D, Schrofelbauer B, Navarro F, Konig R (2003). Species-specific exclusion of APOBEC3G from HIV-1 virions by Vif. Cell.

[ppat-0030015-b014] Chiu YL, Soros VB, Kreisberg JF, Stopak K, Yonemoto W (2005). Cellular APOBEC3G restricts HIV-1 infection in resting CD4^+^ T cells. Nature.

[ppat-0030015-b015] Cen S, Guo F, Niu M, Saadatmand J, Deflassieux J (2004). The interaction between HIV-1 Gag and APOBEC3G. J Biol Chem.

[ppat-0030015-b016] Svarovskaia ES, Xu H, Mbisa JL, Barr R, Gorelick RJ (2004). Human apolipoprotein B mRNA-editing enzyme-catalytic polypeptide-like 3G (APOBEC3G) is incorporated into HIV-1 virions through interactions with viral and nonviral RNAs. J Biol Chem.

[ppat-0030015-b017] Luo K, Liu B, Xiao Z, Yu Y, Yu X (2004). Amino-terminal region of the human immunodeficiency virus type 1 nucleocapsid is required for human APOBEC3G packaging. J Virol.

[ppat-0030015-b018] Khan MA, Kao S, Miyagi E, Takeuchi H, Goila-Gaur R (2005). Viral RNA is required for the association of APOBEC3G with human immunodeficiency virus type 1 nucleoprotein complexes. J Virol.

[ppat-0030015-b019] Schafer A, Bogerd HP, Cullen BR (2004). Specific packaging of APOBEC3G into HIV-1 virions is mediated by the nucleocapsid domain of the gag polyprotein precursor. Virology.

[ppat-0030015-b020] Alce TM, Popik W (2004). APOBEC3G is incorporated into virus-like particles by a direct interaction with HIV-1 Gag nucleocapsid protein. J Biol Chem.

[ppat-0030015-b021] Douaisi M, Dussart S, Courcoul M, Bessou G, Vigne R (2004). HIV-1 and MLV Gag proteins are sufficient to recruit APOBEC3G into virus-like particles. Biochem Biophys Res Commun.

[ppat-0030015-b022] Zennou V, Perez-Caballero D, Gottlinger H, Bieniasz PD (2004). APOBEC3G incorporation into human immunodeficiency virus type 1 particles. J Virol.

[ppat-0030015-b023] Dutko JA, Schafer A, Kenny AE, Cullen BR, Curcio MJ (2005). Inhibition of a yeast LTR retrotransposon by human APOBEC3 cytidine deaminases. Curr Biol.

[ppat-0030015-b024] Suspene R, Sommer P, Henry M, Ferris S, Guetard D (2004). APOBEC3G is a single-stranded DNA cytidine deaminase and functions independently of HIV reverse transcriptase. Nucleic Acids Res.

[ppat-0030015-b025] Schrofelbauer B, Yu Q, Zeitlin SG, Landau NR (2005). Human immunodeficiency virus type 1 Vpr induces the degradation of the UNG and SMUG uracil-DNA glycosylases. J Virol.

[ppat-0030015-b026] Kaiser SM, Emerman M (2006). Uracil DNA glycosylase is dispensable for human immunodeficiency virus type 1 replication and does not contribute to the antiviral effects of the cytidine deaminase APOBEC3G. J Virol.

[ppat-0030015-b027] Chiu YL, Witkowska HE, Hall SC, Santiago M, Soros VB (2006). High-molecular-mass APOBEC3G complexes restrict Alu retrotransposition. Proc Natl Acad Sci U S A.

[ppat-0030015-b028] Kozak SL, Marin M, Rose KM, Bystrom C, Kabat D (2006). The anti-HIV-1 editing enzyme APOBEC3G binds HIV-1 RNA and messenger RNAs that shuttle between polysomes and stress granules. J Biol Chem.

[ppat-0030015-b029] Briggs JA, Wilk T, Welker R, Krausslich HG, Fuller SD (2003). Structural organization of authentic, mature HIV-1 virions and cores. EMBO J.

[ppat-0030015-b030] Briggs JA, Simon MN, Gross I, Krausslich HG, Fuller SD (2004). The stoichiometry of Gag protein in HIV-1. Nat Struct Mol Biol.

[ppat-0030015-b031] Park J, Morrow CD (1993). Mutations in the protease gene of human immunodeficiency virus type 1 affect release and stability of virus particles. Virology.

[ppat-0030015-b032] Chassagne J, Verrelle P, Dionet C, Clavel F, Barre-Sinoussi F (1986). A monoclonal antibody against LAV gag precursor: Use for viral protein analysis and antigenic expression in infected cells. J Immunol.

[ppat-0030015-b033] Forshey BM, Aiken C (2003). Disassembly of human immunodeficiency virus type 1 cores in vitro reveals association of Nef with the subviral ribonucleoprotein complex. J Virol.

[ppat-0030015-b034] Forshey BM, von Schwedler U, Sundquist WI, Aiken C (2002). Formation of a human immunodeficiency virus type 1 core of optimal stability is crucial for viral replication. J Virol.

[ppat-0030015-b035] Fassati A, Goff SP (2001). Characterization of intracellular reverse transcription complexes of human immunodeficiency virus type 1. J Virol.

[ppat-0030015-b036] Blobel G (1971). Isolation of a 5S RNA-protein complex from mammalian ribosomes. Proc Natl Acad Sci U S A.

[ppat-0030015-b037] Stefani G, Fraser CE, Darnell JC, Darnell RB (2004). Fragile X mental retardation protein is associated with translating polyribosomes in neuronal cells. J Neurosci.

[ppat-0030015-b038] Chelico L, Pham P, Calabrese P, Goodman MF (2006). APOBEC3G DNA deaminase acts processively 3' → 5' on single-stranded DNA. Nat Struct Mol Biol.

[ppat-0030015-b039] DiMarzo SJ, Rakoff JS (1986). Intrauterine insemination with husband's washed sperm. Fertil Steril.

[ppat-0030015-b040] Lightfoote MM, Coligan JE, Folks TM, Fauci AS, Martin MA (1986). Structural characterization of reverse transcriptase and endonuclease polypeptides of the acquired immunodeficiency syndrome retrovirus. J Virol.

[ppat-0030015-b041] Cirino NM, Cameron CE, Smith JS, Rausch JW, Roth MJ (1995). Divalent cation modulation of the ribonuclease functions of human immunodeficiency virus reverse transcriptase. Biochemistry.

[ppat-0030015-b042] Shaw-Reid CA, Feuston B, Munshi V, Getty K, Krueger J (2005). Dissecting the effects of DNA polymerase and ribonuclease H inhibitor combinations on HIV-1 reverse-transcriptase activities. Biochemistry.

[ppat-0030015-b043] Shaw-Reid CA, Munshi V, Graham P, Wolfe A, Witmer M (2003). Inhibition of HIV-1 ribonuclease H by a novel diketo acid, 4-[5-(benzoylamino)thien-2-yl]-2,4-dioxobutanoic acid. J Biol Chem.

[ppat-0030015-b044] Schatz O, Cromme FV, Gruninger-Leitch F, Le Grice SF (1989). Point mutations in conserved amino acid residues within the C-terminal domain of HIV-1 reverse transcriptase specifically repress RNase H function. FEBS Lett.

[ppat-0030015-b045] Russell RA, Wiegand HL, Moore MD, Schafer A, McClure MO (2005). Foamy virus Bet proteins function as novel inhibitors of the APOBEC3 family of innate antiretroviral defense factors. J Virol.

[ppat-0030015-b046] Turelli P, Mangeat B, Jost S, Vianin S, Trono D (2004). Inhibition of hepatitis B virus replication by APOBEC3G. Science.

[ppat-0030015-b047] Doehle BP, Schafer A, Wiegand HL, Bogerd HP, Cullen BR (2005). Differential sensitivity of murine leukemia virus to APOBEC3-mediated inhibition is governed by virion exclusion. J Virol.

[ppat-0030015-b048] Wichroski MJ, Robb GB, Rana TM (2006). Human retroviral host restriction factors APOBEC3G and APOBEC3F localize to mRNA processing bodies. PLoS Pathog.

[ppat-0030015-b049] Roy BB, Hu J, Guo X, Russell RS, Guo F (2006). Association of RNA helicase a with human immunodeficiency virus type 1 particles. J Biol Chem.

[ppat-0030015-b050] Kao S, Khan MA, Miyagi E, Plishka R, Buckler-White A (2003). The human immunodeficiency virus type 1 Vif protein reduces intracellular expression and inhibits packaging of APOBEC3G (CEM15), a cellular inhibitor of virus infectivity. J Virol.

[ppat-0030015-b051] Henriet S, Richer D, Bernacchi S, Decroly E, Vigne R (2005). Cooperative and specific binding of Vif to the 5' region of HIV-1 genomic RNA. J Mol Biol.

[ppat-0030015-b052] Zhang H, Pomerantz RJ, Dornadula G, Sun Y (2000). Human immunodeficiency virus type 1 Vif protein is an integral component of an mRNP complex of viral RNA and could be involved in the viral RNA folding and packaging process. J Virol.

[ppat-0030015-b053] Khan MA, Aberham C, Kao S, Akari H, Gorelick R (2001). Human immunodeficiency virus type 1 Vif protein is packaged into the nucleoprotein complex through an interaction with viral genomic RNA. J Virol.

[ppat-0030015-b054] Simon JH, Fouchier RA, Southerling TE, Guerra CB, Grant CK (1997). The Vif and Gag proteins of human immunodeficiency virus type 1 colocalize in infected human T cells. J Virol.

[ppat-0030015-b055] Goncalves J, Shi B, Yang X, Gabuzda D (1995). Biological activity of human immunodeficiency virus type 1 Vif requires membrane targeting by C-terminal basic domains. J Virol.

[ppat-0030015-b056] Kao S, Miyagi E, Khan MA, Takeuchi H, Opi S (2004). Production of infectious human immunodeficiency virus type 1 does not require depletion of APOBEC3G from virus-producing cells. Retrovirology.

[ppat-0030015-b057] Mehle A, Goncalves J, Santa-Marta M, McPike M, Gabuzda D (2004). Phosphorylation of a novel SOCS-box regulates assembly of the HIV-1 Vif-Cul5 complex that promotes APOBEC3G degradation. Genes Dev.

[ppat-0030015-b058] Rosler C, Kock J, Kann M, Malim MH, Blum HE (2005). APOBEC-mediated interference with hepadnavirus production. Hepatology.

[ppat-0030015-b059] Sasada A, Takaori-Kondo A, Shirakawa K, Kobayashi M, Abudu A (2005). APOBEC3G targets human T-cell leukemia virus type 1. Retrovirology.

[ppat-0030015-b060] Newman EN, Holmes RK, Craig HM, Klein KC, Lingappa JR (2005). Antiviral function of APOBEC3G can be dissociated from cytidine deaminase activity. Curr Biol.

[ppat-0030015-b061] Navarro F, Bollman B, Chen H, Konig R, Yu Q (2005). Complementary function of the two catalytic domains of APOBEC3G. Virology.

[ppat-0030015-b062] Stenglein MD, Harris RS (2006). APOBEC3B and APOBEC3F inhibit L1 retrotransposition by a DNA deamination-independent mechanism. J Biol Chem.

[ppat-0030015-b063] Guo F, Cen S, Niu M, Saadatmand J, Kleiman L (2006). Inhibition of formula-primed reverse transcription by human APOBEC3G during human immunodeficiency virus type 1 replication. J Virol.

[ppat-0030015-b064] Noguchi C, Ishino H, Tsuge M, Fujimoto Y, Imamura M (2005). G to A hypermutation of hepatitis B virus. Hepatology.

[ppat-0030015-b065] Rosler C, Kock J, Malim MH, Blum HE, von Weizsacker F (2004). Comment on “Inhibition of hepatitis B virus replication by APOBEC3G.”. Science.

[ppat-0030015-b066] Suspene R, Guetard D, Henry M, Sommer P, Wain-Hobson S (2005). Extensive editing of both hepatitis B virus DNA strands by APOBEC3 cytidine deaminases in vitro and in vivo. Proc Natl Acad Sci U S A.

[ppat-0030015-b067] O'Doherty U, Swiggard WJ, Malim MH (2000). Human immunodeficiency virus type 1 spinoculation enhances infection through virus binding. J Virol.

[ppat-0030015-b068] Adachi A, Ono N, Sakai H, Ogawa K, Shibata R (1991). Generation and characterization of the human immunodeficiency virus type 1 mutants. Arch Virol.

[ppat-0030015-b069] Sakai H, Shibata R, Sakuragi J, Sakuragi S, Kawamura M (1993). Cell-dependent requirement of human immunodeficiency virus type 1 Vif protein for maturation of virus particles. J Virol.

[ppat-0030015-b070] Jenkins Y, McEntee M, Weis K, Greene WC (1998). Characterization of HIV-1 vpr nuclear import: Analysis of signals and pathways. J Cell Biol.

[ppat-0030015-b071] von Schwedler UK, Stuchell M, Muller B, Ward DM, Chung HY (2003). The protein network of HIV budding. Cell.

[ppat-0030015-b072] Esser MT, Graham DR, Coren LV, Trubey CM, Bess JW (2001). Differential incorporation of CD45, CD80 (B7–1), CD86 (B7–2), and major histocompatibility complex class I and II molecules into human immunodeficiency virus type 1 virions and microvesicles: Implications for viral pathogenesis and immune regulation. J Virol.

