# Supplementary material for: Newly Synthesized APOBEC3G Is Incorporated into HIV Virions, Inhibited by HIV RNA, and Subsequently Activated by RNase H
Source: PLoS Pathog. 2007 Feb 9;3(2):e15. doi: 10.1371/journal.ppat.0030015 (PMC1796622; doi:10.1371/journal.ppat.0030015)

Soros et al,
Supplementary Figure 2

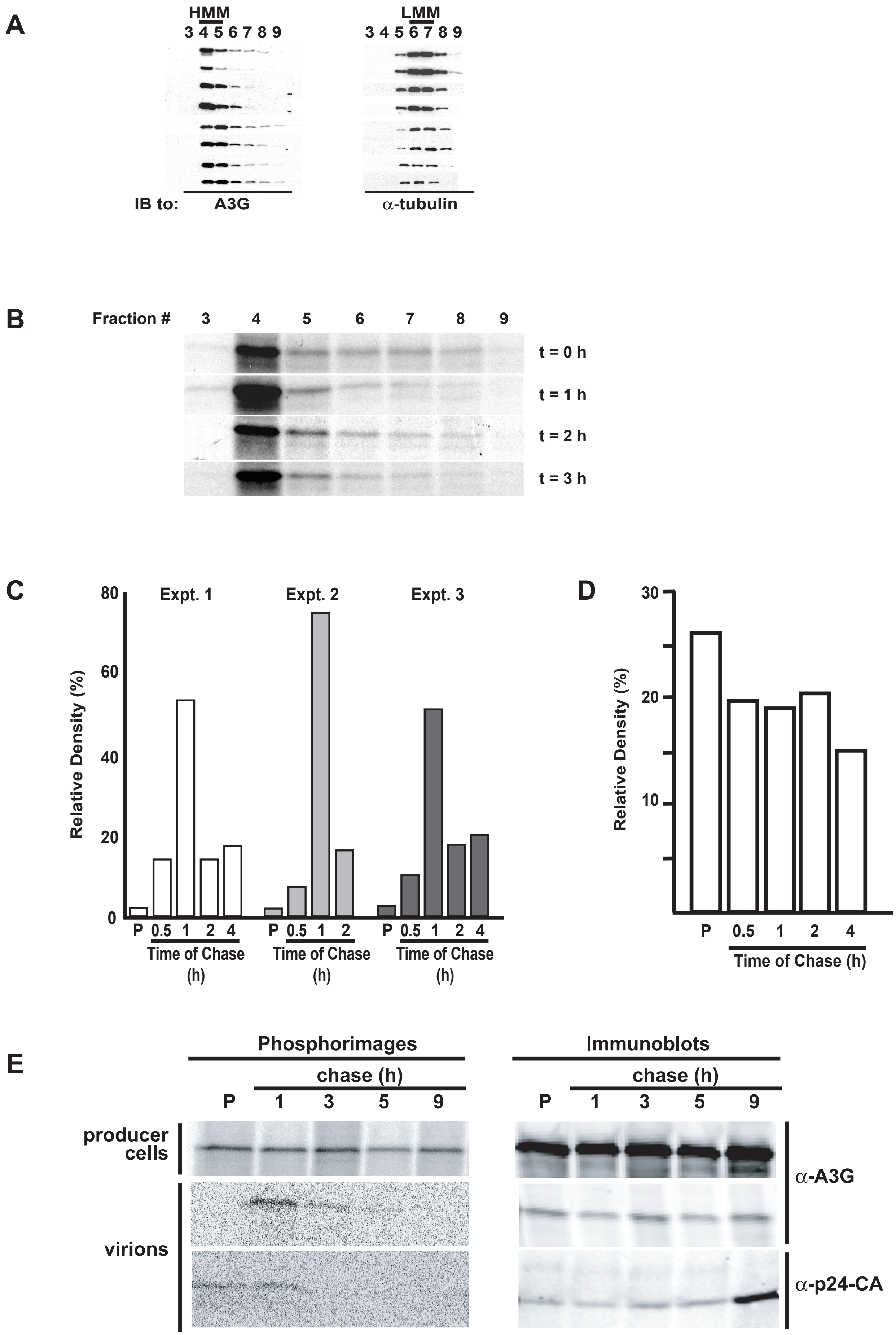

Supplement: Supplementary file 2 [file ppat.0030015.sg002.pdf]
