# Supplementary material for: Newly Synthesized APOBEC3G Is Incorporated into HIV Virions, Inhibited by HIV RNA, and Subsequently Activated by RNase H
Source: PLoS Pathog. 2007 Feb 9;3(2):e15. doi: 10.1371/journal.ppat.0030015 (PMC1796622; doi:10.1371/journal.ppat.0030015)

Soros et al, Supplementary Figure 3

Figure S3

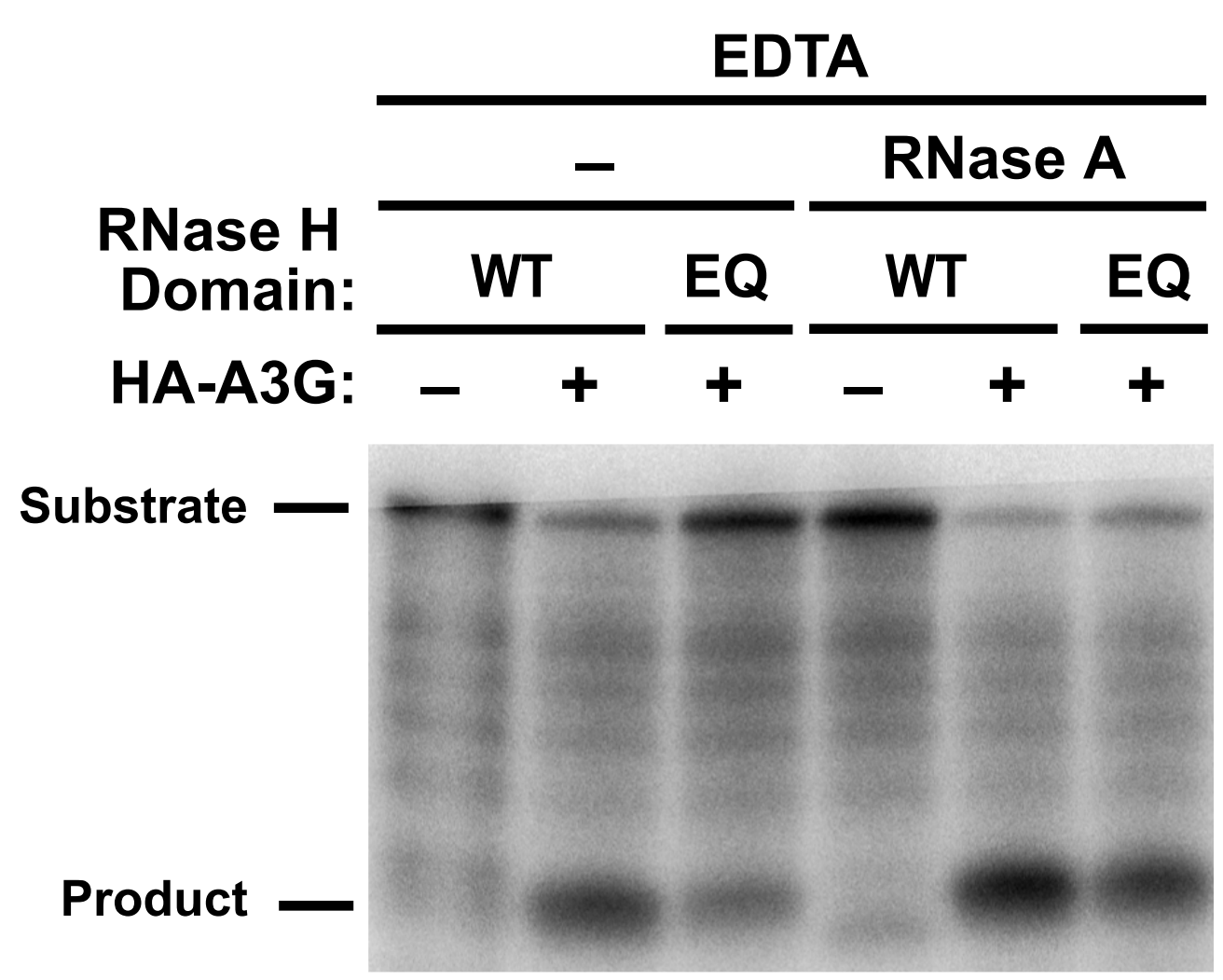

Supplement: Supplementary file 3 [file ppat.0030015.sg003.pdf]
